# The relationship between attitudes to homelessness and perceptions of caring behaviours: a cross-sectional study among women experiencing homelessness, nurses and nursing students

**DOI:** 10.1186/s12905-022-01744-8

**Published:** 2022-05-11

**Authors:** Sophie Nadia Gaber, Andreas Karlsson Rosenblad, Elisabet Mattsson, Anna Klarare

**Affiliations:** 1 Department of Health Care Sciences, Marie Cederschiöld University, Stockholm, Sweden; 2 Department of Women’s and Children’s Health, Healthcare Sciences and E-Health, Uppsala University, Uppsala, Sweden; 3 Department of Medical Sciences, Division of Clinical Diabetology and Metabolism, Uppsala University, Uppsala, Sweden; 4 Department of Neurobiology, Care Sciences and Society, Division of Family Medicine and Primary Care, Karolinska Institutet, Solna, Sweden

**Keywords:** Attitudes, Caring behaviours, Homelessness, Nurse-patient relations, Nursing students, Registered nurses, Women

## Abstract

**Background:**

Women experiencing homelessness have complex and multifaceted healthcare needs and yet they are an underserved population across healthcare services. Nurses are trained to perform an integral role in the provision of equitable healthcare and their attitudes towards homelessness may therefore influence the care that women experiencing homelessness receive. This study aimed to examine correlations between attitudes towards homelessness and caring behaviours, and to test if these correlations differed between the groups of women experiencing homelessness, registered nurses, and nursing students.

**Methods:**

A cross-sectional design using convenience sampling was used to recruit women experiencing homelessness (n = 37), registered nurses (n = 90), and nursing students (n = 138) in Stockholm, Sweden between August 2019 and December 2020. The participants answered two questionnaires: the Attitudes Toward Homelessness Inventory and the Caring Behaviours Inventory-24. Correlations between ordinal variables were calculated using Spearman’s rank correlation ρ. Tests of equality between two independent correlations were performed using a Z-test applied to Fisher’s z-transformed correlations. An advisory board of women with lived experience of homelessness supported the interpretation of the results.

**Results:**

Weak, negative correlations were identified between the Attitudes Toward Homelessness Inventory and Caring Behaviours Inventory-24. The Attitudes Toward Homelessness Inventory mean total scores (SD) were 4.1 (0.6), 4.2 (0.6), 4.1 (0.5) points for the women experiencing homelessness, registered nurse, and nursing student groups, respectively, with the corresponding scores for the Caring Behaviours Inventory-24 being 4.1 (1.1), 5.2 (0.5), 4.8 (0.7) points, respectively.

**Conclusions:**

To promote equitable health for women experiencing homelessness, healthcare providers and nurse educators should consider the role of stigmatising attitudes in relation to caring behaviours.

## Background

Registered nurses (RNs) are trained to perform an integral role in providing equitable healthcare for all people [1]. The basis to this is inclusion health [2], a research, service and policy agenda that seeks to promote health and target health inequities among marginalised communities [3]. Inclusion health is particularly salient for women experiencing homelessness due to their high rates of physical and mental health illnesses [4–7], exposure to violence, abuse, and addiction [4, 6, 8–10], and higher mortality rates than their male counterparts, or the general population [11]. Despite these multifaceted and complex healthcare needs, studies show that women experiencing homelessness are an underserved population across health and social care services [10].

Women experiencing homelessness have reported discriminatory and stigmatising healthcare encounters with nurses [6, 12], which contradicts the ethical imperative of nurses to provide equal care for all people [1, 13, 13, 15]. Nurses who foster caring relationships may positively impact patients’ health and wellbeing by evoking a sense of at-homeness [14, 15]. The concept of at-homeness denotes a sense of being metaphorically at-home, where a person can retreat to feel peace, privacy and safety [14, 15]. Conversely, nurses who violate caring relationships, through negative behaviours and attitudes such as miscommunication and avoidance compound patients’ sense of homelessness and suffering [6, 14]. Thus, it is important to consider both the metaphorical and practical connotations of home and at-homeness in caring relationships between nurses and patients experiencing homelessness.

Nurses develop and foster their caring behaviours through training and clinical experience [16], but they may be susceptible to broader cultural norms and stigmatising attitudes [17–19]. Studies suggest that patients from marginalised communities, including women experiencing homelessness, receive a lower standard of care than the general population, in part due to the clinical biases of health professionals, including nurses [20, 21]. However, research has revealed limited awareness on behalf of health professionals and students regarding their inherent biases or stigmatising attitudes, and the extent to which these may influence their care practice [18, 21, 22]. This raises a potential ethical dilemma about the ways in which nurses’ and nursing students’ attitudes towards women experiencing homelessness may influence their ability to provide equitable healthcare.

Research suggests that nurses and nursing students may be more willing to care for people that they perceive are vulnerable due to societal causes of homelessness, such as housing and job shortages, as opposed to perceived self-inflicted homelessness due to lifestyle or personal causes, such as substance abuse [23–25]. The problem of stigmatising attitudes to women experiencing homelessness may be explained by a lack of awareness of women’s needs and experiences of healthcare, as well as their social and physical context [10, 18]. Crucially, women experiencing homelessness are rarely involved in research [10, 26] and this study adopted a novel approach by asking women experiencing homelessness about their lived experience of healthcare encounters. A study goal was to contribute empirical research from the perspective of women experiencing homelessness themselves to help to address stigmatising attitudes about the causes of homelessness and the ways in which this may impact their interactions with health professionals.

### Theoretical background

To understand the women’s needs and experiences of healthcare it is important to situate their perceptions of caring behaviours and attitudes towards homelessness according to their social and physical contexts. This motivated our use of Urie Bronfenbrenner’s socioecological model to consider the women’s needs and experiences of healthcare in relation to five intersecting, contextual levels: (i) the *microsystem* (i.e., intimate relationships with friends, family and the neighbourhood); (ii) the *mesosystem* (i.e., the interrelatedness of individuals and community organisations); (iii) the *exosystem* (i.e., institutions and workplaces); (iv) the *macrosystem* (i.e., the broader social context including shared cultural norms and attitudes); and (v) the *chronosystem* (i.e., time and changes that occur over the life course) [27–29]. Ideally, a symbiotic relationship exists between individuals and the different contexts; however, the symbiotic relationship and the individual’s sense of agency may be disrupted due to traumatic living situations such as homelessness [29]. There is a knowledge gap regarding nurses’ or nursing students’ attitudes towards homelessness (i.e., the *macrosystem*), and how this relates to the care that they perceive they are *providing* compared with the care that women experiencing homelessness perceive that they are *receiving* (i.e., the *microsystem*). Such knowledge is needed to ensure that women experiencing homelessness receive appropriate levels of care to support their health and wellbeing.

Thus, this study aimed to examine correlations between attitudes towards homelessness and caring behaviours, and to test if these correlations differed between the groups of women experiencing homelessness, registered nurses, and nursing students.

## Methods

### Study design

This study was conducted in Stockholm, Sweden, using a cross-sectional design as part of an overarching project about inclusion health for women experiencing homelessness. The project involved the development of an advisory board of women with lived experience of homelessness in the Spring of 2020 and the board contributed to this study by consulting with the researchers through a workshop focused on the interpretation of the research results. This study adhered to the Strengthening the Reporting of Observational Studies in Epidemiology (STROBE) checklist.

### Participants

The researchers recruited a convenience sample comprised of the following three groups: (i) women experiencing homelessness (n = 37), (ii) RNs (n = 90), and (iii) nursing students (n = 138). The choice of participants was motivated by the study’s aim and enabled a comparison between women experiencing homelessness, as users of healthcare services, as well as RNs and nursing students involved in the provision of healthcare. The sample size was determined based on the aim of being able to detect a 10-point difference, assuming a standard deviation (SD) of 15 points, on the Caring Behaviours Inventory-24 (CBI-24) score between the women experiencing homelessness and the RNs and student groups using the Mann-Whitney *U*-test with a two-sided null hypothesis having α = 0.05 and power = 0.80). Utilising an allocation ratio of 2:1 for nursing students to RNs to allow for the larger pool of available students and attrition rates at 15–20%, the aim was to recruit at least 35 women experiencing homelessness, 35 RNs, and 70 nursing students. The sample size calculation was performed using G*Power 3.1 [30].

#### Women experiencing homelessness

Women experiencing homelessness were recruited from a primary healthcare centre in Stockholm, with support from a research assistant who had experience working with this population. The centre is privately-run with state funding and visits are free of charge. A variety of healthcare services are offered for persons experiencing homelessness and it has close collaborations with social services, primary and psychiatric care, and services for treatment of substance use disorder. Both referrals and walk-in appointments are accepted. The centre receives approximately 14,000 annual visits and provides care for 1300 persons, of which 40% are women. The research assistant approached women in the waiting room of the centre providing services for persons experiencing homelessness. The inclusion criteria were Swedish speaking women with experience of homelessness. Homelessness was defined according to the four categories of the European Typology of Homelessness and Housing Exclusion (ETHOS): (1) rooflessness; (2) houselessness; (3) living in insecure accommodation; and (4) living in inadequate accommodation [31]. The exclusion criteria were women who demonstrated violent and/or abusive behaviours, or who expressed severe anxiety or distress.

#### Registered nurses (RNs)

RNs were recruited at a clinical conference at a private university college with governmental funding in Stockholm. All preceptors (i.e., practice placement supervisors) in Stockholm who receive nursing students from the university college were invited to attend the conference, free of charge. Approximately 110 preceptors attended the conference. Potential participants were approached by researchers who shared information about the study, via a poster board and flyers located near the refreshments area. The information was also emailed to conference attendees who expressed interest in participating but did not have time to sign-up to participate. The inclusion criteria were Swedish speaking and licensed RNs at the time of data collection. The exclusion criterion was RNs who were not clinically active.

#### Nursing students

Nursing students were recruited through a group message on an online learning platform at a university college. The university college is the same location as where the conference was held to recruit the RNs. At the time of recruitment, it had approximately 600 enrolled bachelor nursing students, of which 400 of these would be eligible to participate due to them having completed a clinical placement. The inclusion criteria were Swedish speaking nursing students, who were enrolled in undergraduate nursing education. The exclusion criterion was if the nursing student had not yet experienced a clinical placement.

### Data collection

#### Women experiencing homelessness

Data collection with the women experiencing homelessness began in October and November 2019. After a pause due to the Coronavirus (COVID-19) pandemic, it continued between September and December 2020. The women were provided with verbal and written information and given the opportunity to discuss and ask questions about the study.

With support from the research assistant, the face-to-face data collection occurred at the healthcare centre. Data were collected using pen-and-paper, Swedish versions of the Attitudes Toward Homelessness Inventory (ATHI) [32] and the Caring Behaviours Inventory-24 (CBI-24) instruments [33], in addition to general questions about background characteristics. The women were asked to answer the CBI-24 questions according to how they perceived nurses’ caring behaviours at their latest healthcare visit. As part of a larger research project, the women also answered questionnaires regarding general health, existential health, health literacy, and exposure to violence. The women received a grocery store voucher valued at approximately €10 at the end of the data collection.

#### Registered nurses (RNs)

During October 2019, data were collected with the RNs. The RNs were invited to participate, provided with verbal and written information about the study, and had the opportunity to discuss and ask questions.

Using SurveyMonkey [34], the RNs responded to the ATHI and the CBI-24 instruments, as well as general questions about background characteristics. The RNs were asked to answer the CBI-24 based on how they perceived the caring behaviours of RNs in their clinic. RNs received a lottery scratch card at the end of the data collection.

#### Nursing students

Between August and November 2019, data were collected with the nursing students. The students were provided with information about the study through a message on their learning platform. The message included contact information for the research group and a clickable link to access the questionnaires.

Using SurveyMonkey [34], the students responded to the ATHI and the CBI-24, in addition to general questions about background characteristics. The students were asked to answer the CBI-24 according to how they perceived nurses’ caring behaviours during their latest clinical placement. The students received a lottery scratch card at the end of the data collection.

### Ethical considerations

This study was approved by the Regional Ethical Board in Stockholm, Sweden (Number 2019-021130). Participants were informed that their participation was voluntary and that they may withdraw at any time without any explanation required. Data were collected anonymously and written informed consent was obtained digitally in SurveyMonkey from all participants prior to data collection.

### Instruments

Data were collected using the Swedish versions of the CBI-24 and ATHI instruments. Previous studies have indicated that these instruments are psychometrically acceptable for use in Swedish healthcare research [32, 33]. Psychometric testing of the reliability and validity of the Swedish version of the ATHI indicates acceptable item reliability [32]. The Swedish version of the CBI-24 has good face and content validity, good test–retest reliability, and high internal consistency when administered by RNs to patients in a hospital setting, or when used by nursing students [33].

#### The attitudes toward homelessness inventory (ATHI)

The ATHI is an 11-item instrument measuring multiple dimensions of attitudes towards people in homelessness [35]. The controversy on whether homelessness is caused by personal or societal factors stands at the core of ATHI. Participants answer (score) each item using a 6-point Likert scale (1 = *strongly agree,* 6 = *strongly disagree*). Higher total scores and domain scores indicate more favourable attitudes towards persons experiencing homelessness. The items are categorised into four domains, as follows:

1. *Personal Causation* (PC, 3 items) measures the belief that homelessness is caused by personal deficiencies;
2. *Societal Causation* (SC, 3 items) measures the belief that homelessness is caused by societal factors;
3. *Affiliation* (AFFIL, 2 items) measures attitudes about willingness to associate with persons experiencing homelessness; and
4. *Solutions* (SOLNS, 3 items) measures attitudes regarding viable solutions to homelessness.

#### The caring behaviours inventory-24 (CBI-24)

The CBI-24 is a 24-item instrument which assesses perceptions of caring behaviours among patients and nurses in diverse settings [33, 36]. Participants answer (score) each item according to a 6-point Likert scale (1 = *never*, 6 = *always*). Higher total scores and domain scores correspond to higher perceptions of care provision. The 24 items are categorised into the following four domains:

1. *Assurance* (8 items) assesses how readily available nurses are to patients’ needs and security;
2. *Knowledge and Skill* (5 items) assesses the nurses’ abilities to demonstrate their skills and competence;
3. *Respectful* (6 items) assesses the degree to which nurses show interest and attend to patients; and
4. *Connectedness* (5 items) assesses the nurses’ readiness and willingness to support patients.

### Statistical analyses

Categorical data are presented as frequencies and percentages, n (%), while continuous and ordinal data are given as means with accompanying SDs. Correlations between ordinal variables were calculated using Spearman’s rank correlation ρ, with the strength of correlations classified according to the following for absolute values of the correlations: ≤ 0.20, very weak; > 0.20 to ≤ 0.40, weak; > 0.40 to ≤ 0.60, moderate; > 0.60 to ≤ 0.80, strong; > 0.80 very strong. Tests of equality between two independent correlations were performed using a Z-test applied to Fisher’s z-transformed correlations. All statistical analyses were performed in R ≥ 4.0.0 (R Foundation for Statistical Computing, Vienna, Austria), with P-values < 0.05 considered statistically significant.

## Results

Characteristics of the 37 participating women experiencing homelessness are presented in Table 1, while Table 2 shows the characteristics of the 228 participating nursing students (n = 138) and RNs (n = 90).

**Table 1:** Characteristics of the 37 participating women experiencing homelessness in the cross-sectional study in Stockholm, Sweden, 2019–2020

Variable	Women experiencing homelessness (n = 37) Value
Age, mean (SD)	48.4 (10.4)
Education level, n (%)	
Not finished primary school/other	3 (8.1)
Primary school	11 (29.7)
Secondary school	14 (37.8)
College/University	9 (24.3)
Length of homelessness, n (%)	
≤ 1 year^a^	8 (21.6)
> 1 year but < 5 years	13 (35.1)
5–10 years	11 (29.7)
> 10 years	5 (13.5)

SD, standard deviation. There were no missing values for any of the reported variables

^a^Including one woman stating that she had been homeless “for periods”

**Table 2:** Characteristics of the 228 participating registered nurses and nursing students in the cross-sectional study in Stockholm, Sweden, 2019

	Registered nurses (n = 90)		Nursing students (n = 138)
Variable	Value		Value
Age, mean (SD)	44.9 (11.2)		31.5 (7.8)
Female sex, n (%)	81 (91.0)^a^		127 (92.0)
Years in the profession, n (%)		Semester, n (%)	
≤ 2 years	2 (2.22)	3rd	39(28.3)
3–5 years	10 (11.1)	4th	33(23.9)
6–10 years	21 (23.3)	5th	28(20.3)
> 10 years	57 (63.3)	6th	38 (27.5)

SD, standard deviation. There were no missing values for any of the reported variables

^a^Excluding one individual who answered “Don't want to state”

The women experiencing homelessness were on average 48.4 years old, compared to 44.9 years for RNs and 31.5 years for nursing students. A clear majority (n = 23; 62.2%) of the women experiencing homelessness had a secondary school or college/university education, and most (n = 21; 56.8%) had been homeless for < 5 years. Nine out of ten RNs (n = 81; 91.0%) and nursing students (n = 127; 92.0%) were females, with most (n = 57; 63.3%) RNs having > 10 years of experience in the profession and a slight majority (n =72; 52.2%) of the nursing students being in the 3rd or 4th semester.

Mean scores and standard deviations for the ATHI and CBI-24 are shown in Table 3, separately for the groups of women experiencing homelessness, RNs, and nursing students.

**Table 3:** Mean scores and standard deviations on the ATHI and CBI-24 for the three groups of participants in the cross-sectional study in Stockholm, Sweden, 2019–2020

		Women experiencing homelessness (n=37)	Registered nurses (n=90)	Nursing students (n=138)
Instrument	Domain	Mean (SD)	Mean (SD)	Mean (SD)
ATHI	Personal Causation (PC)	3.3 (1.2)	2.9 (1.0)	3.2 (1.0)
	Societal Causation (SC)	4.9 (0.7)	4.2 (0.9)	4.4 (0.8)
	Affiliation (AFFIL)	4.8 (1.2)	4.2 (1.2)	4.2 (1.2)
	Solutions (SOLNS)	3.4 (1.4)	4.3 (0.9)	4.1 (0.9)
	Total	4.1 (0.6)	4.2 (0.6)	4.1 (0.5)
CBI-24	Assurance (ASR)	4.1 (1.2)	5.3 (0.5)	5.0 (0.7)
	Knowledge and Skill (K&S)	4.5 (1.1)	5.4 (0.5)	5.2 (0.7)
	Respectful (RSP)	4.2 (1.2)	5.3 (0.5)	4.7 (0.8)
	Connectedness (CON)	3.6 (1.3)	4.6 (0.7)	4.1 (1.0)
	Total	4.1 (1.1)	5.2 (0.5)	4.8 (0.7)

Mean scores and standard deviations (SD) on the Attitudes Toward Homelessness Inventory (ATHI) and the Caring Behaviours Inventory-24 (CBI-24) separately for the three groups

Notably, for all ATHI domains, the group of women experiencing homelessness had either the highest (*Personal Causation*, *Societal Causation*, *Affiliation*) or lowest (*Solutions*) mean score, while they had the lowest mean scores for all CBI-24 domains.

Correlations between ATHI and CBI-24 domains are presented in Table 4 separately for the women experiencing homelessness, RNs, and nursing students.

**Table 4:** Spearman’s rank correlation ρ between ATHI and CBI-24 domains for the three groups of participants in the cross-sectional study in Stockholm, Sweden, 2019–2020

Correlated domains		Women experiencing homelessness		Registered nurses		Nursing students		P-values for equality of correlations		
ATHI	CBI-24	ρ	P-value	ρ	P-value	ρ	P-value	WH↔RN^†^	WH↔NS^‡^	RN↔NS^§^
PC	ASR	0.106	0.534	− 0.022	0.836	− 0.168	**0.049**	0.527	0.151	0.284
PC	K&S	0.099	0.559	0.028	0.792	− 0.134	0.117	0.725	0.222	0.236
PC	RSP	0.025	0.884	− 0.009	0.934	− 0.055	0.521	0.868	0.677	0.736
PC	CON	0.154	0.362	− 0.083	0.438	− 0.037	0.666	0.238	0.316	0.738
SC	ASR	− 0.094	0.582	0.005	0.962	0.050	0.557	0.624	0.452	0.742
SC	K&S	0.150	0.375	− 0.089	0.407	0.074	0.386	0.236	0.689	0.235
SC	RSP	− 0.030	0.862	− 0.118	0.269	0.044	0.612	0.661	0.703	0.239
SC	CON	− 0.016	0.928	− 0.095	0.372	0.049	0.572	0.692	0.738	0.295
AFFIL	ASR	− 0.045	0.791	0.118	0.267	0.013	0.883	0.417	0.763	0.440
AFFIL	K&S	0.020	0.905	0.089	0.405	− 0.068	0.426	0.734	0.644	0.252
AFFIL	RSP	− 0.196	0.244	0.084	0.432	0.020	0.819	0.162	0.255	0.640
AFFIL	CON	− 0.159	0.348	0.136	0.200	− 0.015	0.860	0.141	0.450	0.268
SOLNS	ASR	− 0.373	**0.023**	0.061	0.565	− 0.003	0.971	**0.025**	**0.042**	0.638
SOLNS	K&S	− 0.141	0.404	− 0.035	0.746	0.016	0.851	0.595	0.409	0.712
SOLNS	RSP	− 0.202	0.231	− 0.115	0.281	− 0.039	0.647	0.658	0.388	0.581
SOLNS	CON	− 0.335	**0.043**	− 0.113	0.290	− 0.024	0.778	0.244	0.091	0.517
Total	Total	− 0.289	0.083	0.062	0.473	0.009	0.934	0.130	0.061	0.701

AFFIL, Affiliation; ASR, Assurance; CON, Connectedness; K&S, Knowledge and Skill; PC, Personal Causation; RESP, Respectful; SC, Societal Causation; SOLNS, Solutions. Significant P-values are given in bold. Tests of equality between ^†^Women experiencing homelessness (WH) and Registered nurses (RN); ^‡^Women experiencing homelessness (WH) and Nursing students (NS); and ^§^Registered nurses (RN) and Nursing students (NS)

The results of Spearman’s rank correlation ρ analyses indicated a significant negative relationship between the domains *Personal Causation* (ATHI) and *Assurance* (CBI-24) among nursing students (p = 0.049), i.e., the less nursing students believed that homelessness is caused by personal deficiencies, the more they perceived that nurses are readily available to patients’ needs and security.

Furthermore, among women experiencing homelessness the analyses indicated a significant negative relationship between the domains *Solutions* (ATHI) and *Connectedness* (CBI-24) (p = 0.043), i.e., the less women experiencing homelessness believed there are viable solutions to homelessness, the more they perceived that nurses have a readiness and willingness to support patients.

Finally, a significant negative relationship between the domains *Solutions* (ATHI) and *Assurance* (CBI-24) (p = 0.023) was identified among women experiencing homelessness, indicating that the less the women believed there are viable solutions to homelessness, the more they perceived that nurses are readily available to patients’ needs and security. The latter correlation, between *Solutions* (ATHI) and *Assurance* (CBI-24), differed significantly between women experiencing homelessness and RNs (p = 0.025), as well as between women experiencing homelessness and nursing students (p = 0.042). No other correlations differed significantly between any of the three groups.

## Discussion

This study aimed to examine correlations between attitudes towards homelessness and caring behaviours, and to test if these correlations differed between the groups of women experiencing homelessness, RNs, and nursing students. Our results revealed that favourable attitudes to Solutions were significantly negatively correlated to Assurance and Connectedness, among the women experiencing homelessness. This is a somewhat surprising result, which to the authors’ knowledge, has not been discussed in earlier research. This result suggests that among women experiencing homelessness, more favourable attitudes towards viable solutions to homelessness were related to nurses being less readily available to patients’ needs and security (Assurance), or less willing to support patients (Connectedness). To contribute to our study goal and the knowledge gap on research from the perspective of women experiencing homelessness [6], we discussed this result with the advisory board of women with lived experience of homelessness in relation to the different, intersecting contexts of Bronfenbrenner’s socioecological model [27–29]. From the perspective of the members of the women’s advisory board, if more solutions to homelessness are implemented on the *exo-* or *macrosystem* levels, then nurses may be less willing to provide support or be responsive and available to individuals’ needs on the *micro-* or *mesosystem* levels [27–29]. The members of the women’s advisory board summarised the interpretation of the results as follows: the more one believes in society, the less important meetings (i.e., healthcare encounters) are on an individual level. This builds on the concept of at-homeness in earlier research [14].

The correlation between Solutions and Assurance was significantly stronger among the women experiencing homelessness compared with the RNs or nursing students. This result suggests that the women experiencing homelessness perceived that caring behaviours, specifically nurses’ abilities to be readily available to patients, were negatively affected by the attitude that there are viable solutions to homelessness. The women experiencing homelessness perceived this correlation stronger than the RNs and nursing students and this difference may be partially due to different views about what constitutes a *viable* solution. Referring to Bronfenbrenner’s *exo*- and *macrosystem* levels [27–29], housing first (i.e., immediate access to subsidised housing and other supportive services) has been advocated as a solution to address systematic inequities [37, 38]; however, evidence regarding the impact of housing on the physical and mental health of persons in homelessness is inconclusive [37]. The ATHI does not explicitly state examples of viable solutions, or whether the viable solutions are person-centred, and thus one may infer that viable solutions vary between contexts, cultures, communities, and individuals.

To the authors’ knowledge, this study is novel as it is among the first studies investigating microsystem perspectives, in other words asking women experiencing homelessness about their attitudes towards homelessness. In comparison to the RNs and nursing students, the women experiencing homelessness had the most favourable attitudes (i.e., highest mean scores) towards Personal Causation, Societal Causation, and Affiliation; however, they had the least favourable (i.e., lowest mean scores) attitudes towards Solutions. This suggests that the women experiencing homelessness had more favourable attitudes towards homelessness in general than the RNs and nursing students. The women’s more favourable attitudes towards homelessness may be based upon their lived experiences of homelessness, as well as their familiarity with affiliating or associating with other persons experiencing homelessness. The negative attitudes among RNs and nursing students may be partially explained by previous studies that demonstrate that a lack of familiarity with marginalised communities, including persons experiencing homelessness, may exacerbate biases [20, 21, 39]. The negative attitudes contradict the ethical imperative of nurses to provide equal care for all people [1]. Accordingly, the results suggest that there is a need to provide opportunities for RNs in their clinical practice and nursing students in their education to familiarise themselves with underserved populations, such as women experiencing homelessness.

Previous studies indicate that health professionals’ attitudes, values and clinical biases may have a detrimental effect on the provision of care among persons experiencing homelessness [12, 20, 40]; however, our results revealed a more nuanced and varied relationship between attitudes towards homelessness and caring behaviours. The correlations were generally weak or very weak, but a statistically significant negative correlation was found between attitudes towards Personal Causation and the caring behaviour of Assurance among the nursing students. This suggests that among nursing students, more favourable attitudes towards personal deficiencies as a cause of homelessness (Personal Causation) were related to nurses being less readily available to patients’ needs and security (Assurance). In other words, if nursing students perceived that homelessness was self-inflicted due to personal causes, then the caring behaviours towards patients were negatively affected by the attitude. This result corroborates earlier research regarding health professionals’ reluctance to support healthcare needs that they perceive are self-inflicted, and highlights a potential stigmatising attitude which requires attention to avoid being detrimental to the provision of equitable healthcare [23, 24, 40]. Nurses who violate caring relationships with their patients may exacerbate the patients’ sense of homelessness and suffering [14]. Similar to earlier research, this result suggests that it is imperative that RNs [41, 42] and nursing students [24, 43] reflect on their potentially stigmatising attitudes and associated behaviours towards homelessness in order to benefit members of the homeless population. This result aligns with health and social care guideline recommendations that health and social care services should promote engagement among people experiencing homelessness by providing services that are person-centred, empathetic, non-judgemental and that aim to address health inequities [44]. Thus, women experiencing homelessness may benefit from more caring relationships [45] that promote a sense of at-homeness and positively impact their health and wellbeing [14]. This result should be interpreted with caution due to the weak correlation which infers a need for future research to investigate whether other factors, in addition to the attitude that homelessness is self-inflicted, affect nurses’ caring behaviours towards women experiencing homelessness.

### Limitations

The interpretation of the results should be considered in light of the convenience sampling and the data which consequently were not normally distributed. The convenience sampling limited our knowledge regarding the response rate and total number of distributed surveys (i.e., who responded to the surveys in comparison to the number of surveys shared with potential participants), and thus the representativeness of our samples may be questioned. The Swedish language inclusion criteria and the recruitment from a single healthcare centre in the city of Stockholm, the capital of Sweden, are also potential limitations. The healthcare centre lacked EU migrants (including people from the Roma community), who otherwise would have been invited to participate in this study. The focus on a single recruitment site limited the diversity of the sample and the generalisability of the results to less densely populated and rural areas. Since the three groups were mainly comprised of women, future research may benefit from recruiting a more diverse group of participants in terms of sex, age and culture, which may influence attitudes towards homelessness and caring behaviours [40, 46]. Furthermore, we recognise that women experiencing homelessness, like other members of society, differ in needs, capabilities and preferences. The women experiencing homelessness who participated in this study had diverse healthcare needs, including alcohol/drug abuse as well as physical and mental health illnesses. Future research may benefit from focusing on whether specific areas of healthcare needs impact attitudes towards homelessness and caring behaviours.

## Conclusions

To avoid exacerbating a sense of homelessness or suffering, and instead to promote more caring behaviours and equitable health for all, healthcare providers and nurse educators should consider the role of stigmatising attitudes in relation to caring behaviours. Overall﻿, this study revealed weak correlations between the ATHI and CBI-24, and it showed that the women experiencing homelessness perceived the lowest caring behaviours in their healthcare encounters but had the most favourable attitudes towards persons experiencing homelessness relative to the RNs and nursing students. Thus, this study contributes valuable insights about healthcare encounters from the perspective of women experiencing homelessness, a marginalised and seldom heard group in healthcare research and society in general. Consequently, to meet the care needs of this group, it is imperative that RNs and nursing students focus efforts on equitable and respectful caring encounters.

## Data Availability

The datasets generated and/or analysed during the current study are not publicly available due to privacy and ethical restrictions to protect the anonymity of participants but are available from the corresponding author on reasonable request.

## Abbreviations

ATHI: Attitudes Toward Homelessness Inventory
CBI-24: Caring Behaviours Inventory-24
RN: Registered nurse
SD: Standard deviation
